# Brain plasticity-based therapeutics

**DOI:** 10.3389/fnhum.2014.00385

**Published:** 2014-06-27

**Authors:** Michael M. Merzenich, Thomas M. Van Vleet, Mor Nahum

**Affiliations:** ^1^Posit Science CorporationSan Francisco, CA, USA; ^2^Medical Research, Department of Veteran AffairsMartinez, CA, USA; ^3^Department of Optometry, University of California at BerkeleyBerkeley, CA, USA

**Keywords:** brain plasticity, neuroplasticity, computerized training, aging, hemispatial neglect, schizophrenia

## Abstract

The primary objective of this review article is to summarize how the neuroscience of brain plasticity, exploiting new findings in fundamental, integrative and cognitive neuroscience, is changing the therapeutic landscape for professional communities addressing brain-based disorders and disease. After considering the neurological bases of training-driven neuroplasticity, we shall describe how this neuroscience-guided perspective distinguishes this new approach from (a) the more-behavioral, traditional clinical strategies of professional therapy practitioners, and (b) an even more widely applied pharmaceutical treatment model for neurological and psychiatric treatment domains. With that background, we shall argue that neuroplasticity-based treatments will be an important part of future best-treatment practices in neurological and psychiatric medicine.

## Background

In the evolution of treatments of neurological and psychiatric impairments and illness, mainstream medical science has followed two broad paths. One originated with the early Twentieth Century discovery of pharmaceutical agents demonstrated to have powerful, distorting impacts on human neurology (Perrine, 1996; López-Muñoz and Alamo, 2009). Especially from about the middle of the Twentieth Century onward, drug-based medicine has been increasingly strongly supported by technically-sophisticated fundamental neuroscience, which has struggled mightily to describe and define neurological processes and diseases in specific chemical terms, on the path to their chemical manipulation for medical advantage.

The second path, emerging across the same era, began with the insights and discoveries of behavioral scientists and clinicians, who rapidly demonstrated that behavioral abilities could be beneficially modified in patients in need of behavioral adjustment or correction (Boring, 1929; Reisman, 1991). Their cognitive-behavioral therapies have been empirically elaborated in a myriad of ways, to address different levels and aspects of the panoply of symptoms expressed in neurological and psychiatric impairment and disease.

Into the present era, legions of medical professionals predominantly deploy one or the other of these two classes of therapeutic tools to address, in very different ways, the hundreds of neurological and psychiatric disorders that fall within their clinical purview. Both groups see one another as providing an incomplete treatment model. The cognitive (or physical or speech or talk) therapist attempts to correct the distorted expressions of behavior that can so obviously limit the performance abilities of the patient in treatment. Extending a long empirical tradition elaborated by Freud and extended by many others, therapists often also attempt to understand masked biographic neurobehavioral distortions that may still be contributing to current dysfunctions. In another form of treatment, cognitive therapists define the patient's behavioral weakness or limitation as a direct target for correction. If the patient has a negative mood, for example, the therapist works with the patient to improve it via various behavioral strategies; the patient's primary symptom is the direct focus of treatment. If the patient in front of them has a failing memory, to cite another example, the patient is trained to remember—or trained in ways that help them cope with their failing memory. Professionals of a more reductive and chemical persuasion find these strategies to be superficial and necessarily limited by nature. “How can the primary functional expression of a disability or illness be regarded as its cause? How can you expect behaviorally guidance or training to restore a physically and chemically wounded or functionally altered brain in ways that address the underlying causes of impairments or diseases? By what processes can all of that required, detailed chemical and connectional healing occur?”

Their primary answer for addressing those fundamental faults has been the chemical drink or cocktail designed to rebalance or correct or attenuate already-distorted brain chemistry. The behavioral clinician sees such approaches as necessarily crude and limited for addressing the complex neurobehavioral distortions that frustrate the patient in treatment—which, of course, they are. “Treat a patient with a wounded or dysfunctional brain by chemically re-distorting it? How can that provide correction in the key deficits underlying the disorder, when hundreds of chemical processes have been altered as a consequence of the wounding or the disease?” To the behavioral therapist, the exaggeration of the imagined sophistication of the neuro-pharmacological treatment of disease is perhaps only matched by the magnitude of its actual crudity, in neurobehavioral terms.

Beginning about four decades ago, a third vision began to emerge (see Merzenich, 2013, for review). Studies in neuroscience began to elucidate, in progressively more complete form, the neurological origins of behavior. Those studies have now provided us with a first-level understanding of the rules of the processes that govern brain change, both as they account for a progression of the brain in a degrading, “aging,” or distorting—or a strengthening, “rejuvenating,” or corrective neurological direction. This science has also elucidated, in neurological terms, a number of important “failure modes” of the self-organizing brain that have long been given medical labels, like “depression” or “schizophrenia” or “oppositional-defiance disorder” or “Alzheimer's Disease” (see below, and Merzenich, 2013).

Importantly, after a Century of empirical studies by behaviorists trying to understand the origins of neurobehavioral limitations or distortions in humans with neurological impairment or illness, the explosively developing scientific domains of “integrative neuroscience” and “cognitive neuroscience” began to document, with increasing clarity, how and why emergent brain system alterations—expressed by plasticity itself—appear to account for functional human degradation, failure, and disaster. This science has also revealed, with increasing clarity, how limited or distorted neurological processes could be driven, via those same plasticity processes, in strengthening and correcting directions.

Because this evolving science provides a more complete understanding of the origins of—and potentially effective treatment modes for—neurological impairment and illness, we believe that it shall evolve into a foundation science for neurological and psychiatric medicine. Here, the goal is to describe the state of its current scientific development, as a platform for describing what steps can be taken to bring the rapidly developing scientific field of brain plasticity-based therapeutics into medical reality. Our brief review of core principles of neuroplasticity is followed by several practical examples of how the translation of this neurological (and behavioral) science is optimized for therapeutics.

## The science of neuroplasticity

Studies conducted principally over the past 40 years have allowed us to collectively establish the following principles of neuroplasticity:

### The brain is continuously plastic

Not so many years ago, mainstream neuroscience and neurological medicine contended that plasticity was limited to an early childhood epoch—a “critical” or “sensitive period.” We now know that brain remodeling can be induced on a large scale at any age in life (see Swain and Thompson, 1993; Merzenich and de Charms, 1996; Merzenich, 2001, 2013; Weinberger, 2004; Gilbert et al., 2009). What differs as a function of age is the way in which the brain regulates plasticity. In the very young brain, almost all inputs continuously engage competitive plasticity processes. In older brains, plasticity is regulated as a function of behavioral context and outcomes.

### In the older brain, a context- and outcomes-dependent release of neuromodulators from subcortical limbic system nuclei enable and trigger brain change

In the perinatal and early-childhood “critical period,” plasticity-enabling conditions are always “on.” In the older child and adult brain, changes in the control of the release of “neuro-modulatory neurotransmitters”—and in the properties of the receptors in the brain that govern their actions—enable the older brain's moment-by-moment control of change; it is permitted *only* when the specific contextual conditions that enable or trigger plasticity are met, with changes arising under those special contextual-enabling conditions “saved” (driving enduring changes in connection strengths) as a function of behavioral outcome (e.g., see Merzenich, 2001, 2013 for reviews).

For example, under conditions of focused attention, any stimulus excites acetylcholine (ACh) releasing neurons in the basal nucleus of Meynert (Richardson and DeLong, 1990; Sarter et al., 2001, 2006). In the cortex, ACh inputs positively enable plasticity by (a) selectively amplifying only anticipated (“selectively attended”) and (b) selectively weakening non-anticipated inputs—including those at any given cortical location that may have most effectively excited neurons before learning-induced changes were initiated (Sarter et al., 2006; Froemke et al., 2007). By this action, brain circuits enable plasticity by advantaging input strengths for those specific activities that the brain can gain in ability by changing to, and disadvantage behaviorally non-contributing inputs that they shall change from.

As a second example, noradrenaline (NA) releasing neurons in the locus coeruleus (LC) (and in nucleus accumbens and amygdala) broadly amplify neuronal activity, increasing the general level of excitability (arousal, or baseline level of attention) in sub-cortical and cortical structures in any closely-attended context (for example, in stimulus- or goal-seeking or other “motivated” states) (see, e.g., Aston-Jones and Cohen, 2005; Sara, 2009; Sara and Bouret, 2012). NA is also released to selectively amplify the activities evoked by unexpected (novel) input (Aston-Jones et al., 1999), conferring special powers for the representation of “surprising” inputs or activities for driving enduring representational change.

Dopamine (DA) releasing neurons in the ventral tegmental area and substantia nigra are highly specific plasticity enablers (see, e.g., Bao et al., 2001, 2003; Winder et al., 2002; Lisman et al., 2011). They are excited when the brain receives—or first predicts the occurrence of—a hedonic input (reward), or when the brain achieves or first predicts behavioral success (for which it “rewards itself”) in a learning cycle (Schultz, 2007). With their release, inputs that “predict” that reward (i.e., are highly correlated with its occurrence) are selectively strengthened; competitive inputs uncorrelated with reward prediction arriving in a short post-reward epoch are selectively weakened (see Ahissar et al., 1992; Bao et al., 2003).

We now have a first-level understanding of the “rules” that control the release and the actions of these (and other) neuromodulators in learning, and of the modulator-specific ways that they nuance brain changes in experience and learning.

It should be noted that this crucial neuromodulatory machinery, controlling learning and memory abilities throughout life, is also plastic (Nakamura and Sakaguchi, 1990; Sara and Segal, 1991; Steiner et al., 2006; Smith et al., 2011; Zhou et al., in review). The strengths, selectivity, and reliability of its actions can be significantly improved via intensive training in most individuals with neurological or psychiatric impairment or disability.

### Many aspects of the neurological representations of inputs and actions can be modified by appropriate neurobehavioral training

In early studies of plasticity processes, we and others conducted studies designed to reveal which aspects of the representations of inputs or actions could be improved by training, under the right contextual conditions, in the adult brain (see Merzenich, 2013, for review). It was quickly shown that we could change the selectivity of neuronal responses (i.e., receptive field sizes); the memberships of competing populations of neurons (“mini-columns;” Buxhoeveden and Casanova, 2002) that represent those selective inputs; the detailed representation of stimulus magnitudes; stimulus modulation rates; successive-signal segmentation and integration (“masking;” “sampling rate”); stimulus duration and inter-stimulus interval resolution and estimation; spectrotemporal or spatiotemporal stimulus complexity; stimulus sequencing; stimulus source location or identification; signal-to-noise conditions for stimulus representation, and response reliability—among other parameters of inputs (see, e.g., Merzenich and de Charms, 1996; Gilbert et al., 2009; de Villers-Sidani et al., 2010; Merzenich, 2013). In the domain of action, we could similarly drive improvements in response reliability; response speed; replication of timing in responding; response accuracy; response sequence reconstruction; and response fluency; among other parameters of action control. Many other scientists have extended these studies to demonstrate plasticity in other perceptual, working memory, associative memory, selective attention, sustained attention, distractor suppression, among other functional neurological abilities.

It should be noted that these studies have also shown that all of these same (and many other) aspects of the neurological representations of inputs and actions can be driven by training, just as easily, in a degrading direction (e.g., see Merzenich and Jenkins, 1993; Zhou et al., 2011)—again by the action of normal brain plasticity processes.

### The primary plastic change is in the strengths of connections (synapses) in brain circuits

Neuroplasticity research has extensively documented the phenomenology of—and the cellular and molecular processes underlying—the plastic remodeling of the “wiring” in brain circuits. The central governing rule was postulated by the Canadian psychologist Donald Hebb in the 1940's: “What fires together, wires together” (Hebb, 1949). This coincident-input-dependent co-strengthening of synaptic connections occurring moment by moment in time in a learning context is achieved through both a multiplicity of physical changes in synapses that amplify connection strength, and by synaptogenesis. The magnitude of such changes under near-optimum learning contexts can be remarkable: a large proportion of synapses in any directly engaged cortical zone (commonly, many millions to billions of synapses) are altered in their connection strengths as you acquire any significant skill or ability (e.g., Kleim et al., 2002). As we master any skill or ability through experience or progressive learning, these changes in brain circuitry result in the specialization of the brain as a master receiver and master controller of all of the inputs and actions supporting that mastery.

Nonetheless, the same processes that confer growth in synaptic power for inputs that contribute to neurobehavioral advance are also driven backward, for other non-behaviorally-contributing synapses, in a synaptic weakening and synapse elimination direction (see below). This “normalization” of collective synaptic input power has been extensively studied in other experimental models by depriving neurons of a major source of their inputs; in that event, synaptic strengths are rapidly adjusted to sustain neurons within a narrow electrical potential window that assures their ongoing functional viability (Horng and Sur, 2006; Cooper and Bear, 2012; Feldman, 2012).

### Plasticity controls functional reliability via its generation of neuronal cooperativity

Through Hebbian network plasticity, the extensively cross-wired neurons in the cerebral cortex also strengthen their connections with their nearest neighbors. When the brain is engaged behaviorally, inputs that are activated nearly simultaneous in time strengthen together, increasing their cooperativity to generate more salient (i.e., more collectively powerful, more reliable) responses. That plasticity-driven growth in local “teamwork” is a critical aspect of the improvement in the processing of information supporting learning-based advances in behavior (see Edelman, 1987; Merzenich and Jenkins, 1993; Merzenich and de Charms, 1996; Merzenich, 2013).

Learning-driven increases in neuronal response coordination are a primary determinant of the feed-forward power of any plastically strengthening cortical process. Cortical neurons at all “higher” system levels are integrators operating with short time constants. Their plasticity processes are also coincident-input dependent. The greater the coordination of neurons in the lower levels of the network that feeds them, the greater their selective powers and selectivity, and the greater the power of that input to drive plastic remodeling at higher system levels. Moreover, at the “top” of our great brain systems, coordination of activity is a primary determinant of the ability of cortical networks to sustain the reverberant activities that are selective for behavioral targets or goals (i.e., working memory) (see Wang et al., 2004; Compte, 2006). The strengths of these key plasticity-gating processes “at the top” are crucially dependent upon the strengths, i.e., collective coordination, of the inputs that feed them.

### Neuroplasticity drives changes that broadly remodel the physical brain

Changing synaptic strengths and synaptogenesis involves complex physical change processes resulting from changes in genetic expression of several hundred well-described molecular processes. At the same time, there are many other physical changes induced by brain plasticity processes, collectively involving several *thousand* known molecular processes. The physical processes of neurons (the receiving “dendrites” and their synaptic “spines;” the transmitting “axons” and the elaboration of their terminal arbors; the distributions of collateral axons that richly interconnect neurons within cortical networks; the processes and cell-to-cell contacts of closely coupled non-neuron glial cells) can be plastically altered on a large scale, resulting in changes in cortical thickness, neuropil volumes, and cortical area and subcortical nucleus volumes (see Merzenich, 2013, for review). Specific cell types can shrink or greatly expand in size, and can be greatly metabolically reduced or invigorated—all expressed through easily-documented, controlled, plastically-induced physical change. The insulating myelin can be thickened—or thinned—under plastic control (de Villers-Sidani et al., 2010; Zhou et al., 2012). Chemical factors controlling the health and vigor and operational characteristics or brain systems, or contributing to the regulation of plasticity itself—including “trophic factors,” transporters, excitatory, inhibitory and neuro-modulatory neurotransmitters and receptors are all altered physically, when the brain advances, or retreats, by the action of adult neuroplasticity processes (Merzenich, 2013).

### Brain systems account for our explicit behaviors

A large body of science has now shown that our expressive behaviors are a product of complex, multi-level recurrent networks (for further discussion and review, see Merzenich, 2013; Nahum et al., 2013c). In these networks, information is represented with greatest resolution in detail in place, feature, and time at lowest network (“system”) levels. At successively higher levels, there is an integration of representation to progressively more complex objects, relationships and actions, as they apply in the “real world.” At the “top” of brain systems, those most-completely-integrated neurological representations generate enduring neural activity that is selective for their representation. That persistent reverberant activity, providing the neurological basis of working memory, can be sustained in the human brain for tens of seconds to minutes of time (see Goldman-Rakic, 1995; Compte, 2006; Merzenich, 2013). It is important to understand that representational information is continuously fed backward from this highest (and from all other) level(s). In these recursive recurrent networks, the operational levels contributing to the representation of any aspect of input or action in brain systems are inseparable; in other words, all explicit behaviors are a product of the *system*. Therefore, when evident behaviors are distorted or impaired, as they are in the many ways that define the fundamental deficits and nuances of different specific neurological and psychiatric clinical indications, we necessarily target neurological renormalization at all system levels when designing therapeutic training programs.

### In a brain system, plasticity is controlled “from the top”

Recent neuroscience studies have shown that through recursive re-entrant feedback (see Edelman, 1987; Grossberg, 2013; and Merzenich, 2013, for review), the representation of information “at the top” of our forebrain processing systems selectively enables plastic changes contributing to the progressive behavioral success of brain systems (see Hochstein and Ahissar, 2002). At highest system levels, behavioral targets are held, as described, via sustained target-specific activities, in working memory. That sustained persistently reverberant activity is projected backward down to “lower” system levels, where it positively enables plasticity for any fed-forward activity that can potentially contribute to a progressively improving resultant. Scientists often call the opening of this window that controls, through this top-down biasing, what the brain can change to, a “selective attention” process. In fact, “working memory” and “selective attention” can be considered to be two descriptors of the same persistent reverberant activity-based representation/feedback process (see Fuster, 2008). This process also provides the neurological basis of the brain's predictive, associative memory, sequencing construction, and syntactic powers.

The neurological processes by which feedback “from the top” biases plasticity in learning at all lower network levels are now understood, at a first level. Biasing is achieved, neurologically, by dis-inhibition processes in cortical networks controlled by convergent modulation “from the top” on the one hand (through a selective attention process), and from a cholinergic subcortical input source engaged under conditions of focused attention, the basal nucleus of Meynert (see Sarter et al., 2001, 2006; Froemke et al., 2007; Weinberger, 2007; Carcea and Froemke, 2013; also see Zhou et al., 2010), on the other hand.

Ahissar and Hochstein (Hochstein and Ahissar, 2002; Ahissar et al., 2009) have described this feedback plasticity-enabling biasing, in psychological science terms, as “the reverse hierarchy theory.” According to this perspective, the brain holds a model of a behavioral event or training goal in working memory; that model, fed back to lower system levels, selectively amplifies activities (through dis-inhibition) that the brain can change *to*, as it progressively sharpens and refines, through learning, the resultant—its working memory-sustained models.

### Plasticity engages both synaptic strengthening and synaptic weakening processes

Fundamental studies of plasticity mechanisms have shown that every brief change cycle invokes a synapse-strengthening moment (e.g., strengthening all inputs whose coordinated actions moment by moment in time are correlated with a positive behavioral outcome), followed by a synapse-weakening moment (e.g., weakening all inputs occurring within a brief, following epoch of time) (Dan and Poo, 2006; Cooper and Bear, 2012). As noted earlier, this synapse weakening can be viewed as an electrically homeostatic process that contributes to the ongoing weakening of behaviorally non-meaningful intrinsic activities or inputs—that is, to a normalization of internal or background external (environmental) noise.

Viewed from another perspective, plasticity processes can be viewed as continuously competitive. Through these two-way plasticity processes, neurons in coupled “mini-columns” are continuously competing with their neighbors for the domination on neurons on their mutual boundaries (see Merzenich and Jenkins, 1993; Merzenich, 2013). By giving one coupled group the competitive advantage over their neighbors, it is easy to expand their team a 1000-fold—or, if they are a competitive loser, to reduce its “membership” many times over. By giving any one source of input a competitive advantage or disadvantage, the territory it comes to dominate in the brain can be dramatically enlarged, or contracted; every moment of gain for the “winner” is a moment of loss for “losers.” By these two-way processes, one can easily both refine (for some inputs) and degrade (other inputs)—even the most-fundamental aspects of representation of visual or auditory or somatosensory inputs in the adult brain.

### At least most (possibly all) plasticity-induced changes are, by their nature, reversible

Plasticity engages fundamentally reversible neurological change processes. We have conducted a number of studies that have demonstrated that neuroplasticity follows Hebbian principles: the representations of inputs and actions are competitively sorted on the basis of the temporal distributions of inputs (Merzenich and Jenkins, 1993; Merzenich and de Charms, 1996). Following these principles, it is just as easy to degrade the brain's processing abilities as it is to strengthen or refine them. In the designs of therapeutic training regimes, the Hebbian “rule” must be considered, to assure that training-driven changes are always in the positive, strengthening, recovering, re-normalizing direction.

We have recently conducted a number of studies in animals that show that plasticity processes are very broadly reversible. For example, after documenting many aspects of the function, anatomy and chemistry in the brains of aged vs. young adult animals, it was shown that every measure differed markedly (de Villers-Sidani et al., 2010; de Villers-Sidani and Merzenich, 2011; Mishra et al., under review). In the aged rats' auditory cortices, time and space constants were longer and greater; response selectivity was poorer; reliability of sound feature representation was poorer; response correlation was weaker; the neuron populations representing sensory inputs were less strongly coupled, operating with far weaker cooperativity; inhibitory processes controlling “top-down” modulation were weaker; local and long range connections were more poorly myelinated; level-to-level (system) coordination (in gamma and theta frequency ranges) was less sharply localized and more weakly persistent; representational topographies were degraded; trophic factors contributing to metabolic and physical maintenance and plasticity were only weakly expressed; the normally strong adaptation to repeated identical stimuli and responses to unexpected stimuli against a continuous or repeated background were sharply reduced; the strong suppression of non-attended distractors was reduced; receptor subunits for inhibitory and excitatory processes were altered in a degrading direction; and the modulatory control processes controlling plasticity were all more weakly operating in very old vs. prime-of-life animals. After recording these manifold, significant differences between aged and young rats' brains, animals were intensively behaviorally trained in operant tasks to determine which of these operational characteristics of the brain could be “rejuvenated.” Somewhat to our surprise, with training limited in these aged rats to approximately 1 h/day for about 1 month, all of these degraded operational and physical-chemical characteristics of the aged brain could be substantially if not completely restored to a “youthful” state, in aged animals (de Villers-Sidani et al., 2010; de Villers-Sidani and Merzenich, 2011; Mishra et al., under review).

Given its reversible nature, plasticity processes can just as easily be engaged in a young prime-of-life animal in ways that drive their brains in an increasingly uncorrelated pattern activity (as seen in aged animals). That has also been achieved for the auditory brain by a simpler environmental exposure strategy. By housing young, vigorous adults in an environment of non-correlated noise (believed to increase the level of internal noise in the hearing brain) for a period of several weeks, *all* of the functional and physical characteristics of the machinery of the brain noted above altered *as if* the animal had advanced over those several weeks to an “old age” status (Zhou et al., 2012; Kamal et al., 2013).

Because these reversible change processes can drive neurological changes in either an advancing or degrading direction, driving the processing and physical characteristics of the brain rapidly “forward” to simulate aging is equivalent to driving the animal backward in age: The physical and functional properties of the brain near the end of life closely correspond to those same characteristics in the brain recorded near the beginning of life (Zhou et al., 2012). That conclusion is supported by documenting the operational and physical characteristics of the machinery of the brain in very old and very young animals: they closely match one another. It is also manifested by the fact that key accelerated changes leading to “premature aging” achieved by noise exposure or by “negative” training, carried forward far enough, similarly result in the re-opening of the “critical period” (Zhou et al., 2012).

### The neuroscience of brain plasticity provides new insights into the origins of the expressions and natures of acquired neurological and psychiatric impairment and “disease”—and in how to drive “corrective” changes in impaired brains via intensive training

From the study of all of these complex aspects of brain change, neuroscientists have defined the “rules” that govern them, in the terms of the brain processes that account for these aspects of brain change. We now understand necessary and optimal conditions for driving positive changes in most dimensions of brain processing, as well as the behavioral functionality that they account for. This rule-based training represents an important refinement of the more-empirically based development of “cognitive training” program designs, in several important respects. First, with this understanding, we can more directly target neurological (not merely behavioral) improvement or re-normalization. Second, this science is progressively resolving long-standing arguments about “best practices.” There *is* a best way: following the brain's rules for learning-based remodeling.

Neuroplasticity research, with related studies in fundamental and integrative neuroscience conducted in animal and human models, has provided us with a new level of understanding of the neurological bases of representation of behavior. It has shown us, at a deeper level, the natures of the neurological distortions that underlie function impairment, loss, or dysfunction, specifically defined in neurological (not limited to descriptive behavioral) terms. Fundamental recovery, which addresses the central deficit underlying the disorder, must result in neurological improvement or restoration. With this rapidly growing science, we can potentially extend our targeting of neurological dysfunction to the more elemental processes in brain systems that account for behaviorally expressed impairments.

To understand how this differs from the currently predominant approaches to neurological and psychiatric medicine, consider two simple examples: First, scientists have studied alterations in many disease states by documenting which genes are up-regulated—and which are down-regulated—in specific diseases (Gilman et al., 2012; Calciano et al., 2013; Fass et al., 2014; among several hundred citable examples). One of their goals has been to determine what specific change processes could account for the disease's emergence, on the path to the potential pharmaceutical treatment of the illness. With remarkable repetition, study after study has recorded: (1) several to many hundreds of genes that are significantly up-regulated and down-regulated in the disease or condition; and (2) a broad overlap in this pattern of change for brains studied from patients with even strikingly different clinical indications (like schizophrenia or autism or Alzheimer's disease or multiple sclerosis). For example, genetic variation in encoding brain derived neurotrophic factor (BDNF) has been implicated in neuropsychiatric disorders such as Alzheimer's disease, affective disorders, schizophrenia, and substance dependence (e.g., Zhang et al., 2006).

From a brain plasticity perspective, these gene chip results are unsurprising: any struggling brain undergoes broad-scale “plastic” revision. It is highly probable that most of the recorded changes in gene expression are a reflection of plastic remodeling; in the face of growing “noise” in neurological processes, the brain plastically adapts to retain some level of functional control (see Merzenich, 2013). Given the chemical complexity of these changes contributing to disease expression, no single drug or limited drug combination, and no simple training of an explicit behavioral ability(ies) can be expected to drive the myriad of coupled plastically-adjusting processes to correction. On the other hand, animal studies indicate that it may be possible to achieve broad-scale “reversals” in targeted brain systems via relatively simple intensive training programs repertoires.

To cite a second example, cognitive psychologists have identified the ability of brain systems to sustain reverberant activities representing specific information “held in working memory” as a primary cause of many problems, extending from ADHD through schizophrenia to mild cognitive impairment. Their predominant treatment solution has been to directly exercise this failing faculty; medically useful gains are achieved by such training (Klingberg, 2010). However, as in any form of explicit skills training, benefits do not broadly generalize to other task domains that engage working memory in real-life behaviors (Melby-Lervåg and Hulme, 2013; Rapport et al., 2013).

A brain plasticity perspective addresses this kind of problem from a deeper level of understanding. “What is the cause of the inability of the brain to generate strong, persistent activities? How and why is the system not generating the highly correlated feed-forward inputs and/or neuromodulatory inputs both known to be crucial for its genesis?” From that perspective, broader training designed to increase the salience (correlated power) of representation of the details of inputs and actions at every system level and the assured or corrected function of neuromodulatory contributors to working memory processing would be deemed to also be important for achieving stronger far-transfer training impacts (see, for example, Strenziok et al., 2014).

## Advances in cognitive neuroscience have also profoundly changed the therapeutic landscape

A rapid expansion and elaboration of human brain recording and imaging studies have paralleled the phenomenal growth of neuroplasticity-related neuroscience. An increasing number of those studies document aspects of training-driven plasticity itself. Still, the primary focus of this research has been the mapping of patterns of activity in brain systems that account for specific human abilities. That science provides a basis for defining alterations in abnormal brain systems accounting for almost every class of functional impairment or “illness.” This science has been limited with respect to the completeness with which it records abnormality in specific neurological-process terms. Still, it provides great insights into the origins of and the bases of behavioral expressions of every important neurologically-based clinical indication—and is a crucial source of information for our designs of impairment- and disease-targeted plasticity-based therapeutic programs. It also provides an increasingly definitive basis for documenting therapeutic outcomes, where the primary goal in therapy is shifting beyond behavioral improvement to the potential re-normalization of dysfunctional brain systems. Examples of how we apply this key source of information in program designs are described below.

## Translating neurological (and behavioral) science into optimized therapeutics

Based on the neuroscience of brain plasticity, therapeutic training strategies have been created that target a growing number of clinical conditions. Here, three examples illustrate how these programs are created and validated for therapeutic use.

### Targeting neurological and behavioral impairments in schizophrenia

A growing body of literature points to pervasive neurocognitive and social cognitive impairments as fundamental aspects of the expression of schizophrenia (e.g., Cirillo and Seidman, 2003; Brewer et al., 2005; Keefe et al., 2006; Eastvold et al., 2007; Becker et al., 2010; Kim et al., 2011). Deficits in perception, speed of processing, working memory, attention, executive function, social cue perception, and social and action control are recorded even before illness onset, and are associated with poor functional, societal and occupational outcome (Green et al., 2000, 2012; Edwards et al., 2001; Lencz et al., 2006; Niendam et al., 2006; Seidman et al., 2006; Simon et al., 2007; Chan et al., 2010; Kohler et al., 2010; Billeke and Aboitiz, 2013). Because the perceptual, cognitive and social cognitive deficits are generally dissociated from psychotic symptoms in schizophrenia, they are not significantly ameliorated by antipsychotic medication (e.g., Goldberg et al., 2007; Green, 2007). Specifically, second-generation dopamine-agonists antipsychotic did not show any advantage over first-generation agents in treatment of cognitive deficits in schizophrenia (Keefe et al., 2007). Similar negative effects for cognitive deficits in schizophrenia have been reported for clinical trials involving glutamate-related or serotonergic agents (see Goff et al., 2011, for recent review).

At the same time, we hypothesize that these weaknesses in perceptual and cognitive processing underlie the catastrophic breakdown of working memory operations, which are at the heart of psychoses genesis (see Merzenich, 2013). By that interpretation, training that improves the neurological abilities that contribute to working memory and neuro-modulatory system functionality in these great brain systems should have substantial prophylactic power in at-risk individuals.

From a neurological perspective, schizophrenic brains are: poor signal resolvers, operate sluggishly, struggle to generate sustained activities supporting top-down (working memory, selective attention, associative memory, predictive) processes in prefrontal cortex (Minzenberg et al., 2009), and in frontal, posterior parietal and inferior and medial temporal areas (Heckers, 2001); have distortions in language, visual, source-reference and other operations related to psychotic symptoms (Modinos et al., 2013); have impairments in social cognition that greatly impact quality of life (see Couture et al., 2006); and have changes in fundamental neuronal processes that we associate (along with working memory degradation) with very noisy brain system processing (e.g., Hinkley et al., 2011).

Perceptual, cognitive, social, and motor control deficits along with modulatory system abnormalities are obvious, important targets for treatment in schizophrenia. From a brain plasticity perspective, fundamental neurocognitive recovery shall require brain system remodeling: high-speed, high-fidelity processing with systems engaged by progressively more complex inputs and more difficult challenges should be combined with more-explicit cognitive re-training to achieve system re-normalization. As a part of a brain plasticity-based recovery strategy, it is important to re-normalize neuro-modulatory processes controlling plasticity itself. Core deficits expressed in the illness—the usually-severe degradation of working memory processes and magnified levels of arousal and intensity—are attributable in part to abnormally high levels of expressions of dopamine and NA in these individuals (Tost et al., 2010). By that dysregulation, the contribution of these systems to plasticity itself can be significantly altered—with further distortions induced by the psychoactive drugs that target the expressions of these neuro-modulators to ameliorate this self-poisoning.

Our computerized cognitive training programs are designed to drive the brain of the schizophrenic patient broadly in the normal-ward direction, in: auditory/aural speech, visual, social cognition, executive, social, and action control domains, attention and focus, and in neuro-modulatory system control domains. In these targeted brain system, training extends from low-level perceptual processing to higher-level working memory, attention and executive and motor control processes. Subjects are trained to refine representational fidelity and operate at speed, in ways designed to reduce internal brain noise and restore more normal physico-chemical processing. Training of implicit abilities is designed to assure that all fundamental processing abilities are refined in ways that support more reliable and more sophisticated explicit operations. All exercises are progressive and adaptive, adjusting in difficulty to assure a continuously successful-but-challenging level of ongoing training. The generalization of gains to all processing that engages the targeted systems is a universal program goal.

Specific attention has been given in our exercise suite to training of social cognition, which has been recently pointed out as a particularly important target for intervention in schizophrenia. Specifically, social cognition deficits have been directly linked to poor functional outcome in schizophrenia (Couture et al., 2006) and have been found to underlie critical factors affecting daily living in schizophrenia, such as occupational status, community functioning, independent living skills, and quality of life (e.g., Couture et al., 2006; Bell et al., 2009; Fett et al., 2011). Our Social cognitive training (“SocialVille;” Nahum et al., 2013a,b) deploys socially-relevant stimuli in tasks that target affect perception, social cue perception, theory of mind and attributional style. The SocialVille exercises require progressively more complex discrimination and identification of socially-valid stimuli, while again driving progressive improvements in processing speed, working memory, and attention control. We have recently successfully completed a pilot feasibility study of SocialVille in early-phase schizophrenia patients, who completed training remotely from home using internet-connected laptops (Nahum et al., 2014).

**Figure 1:**
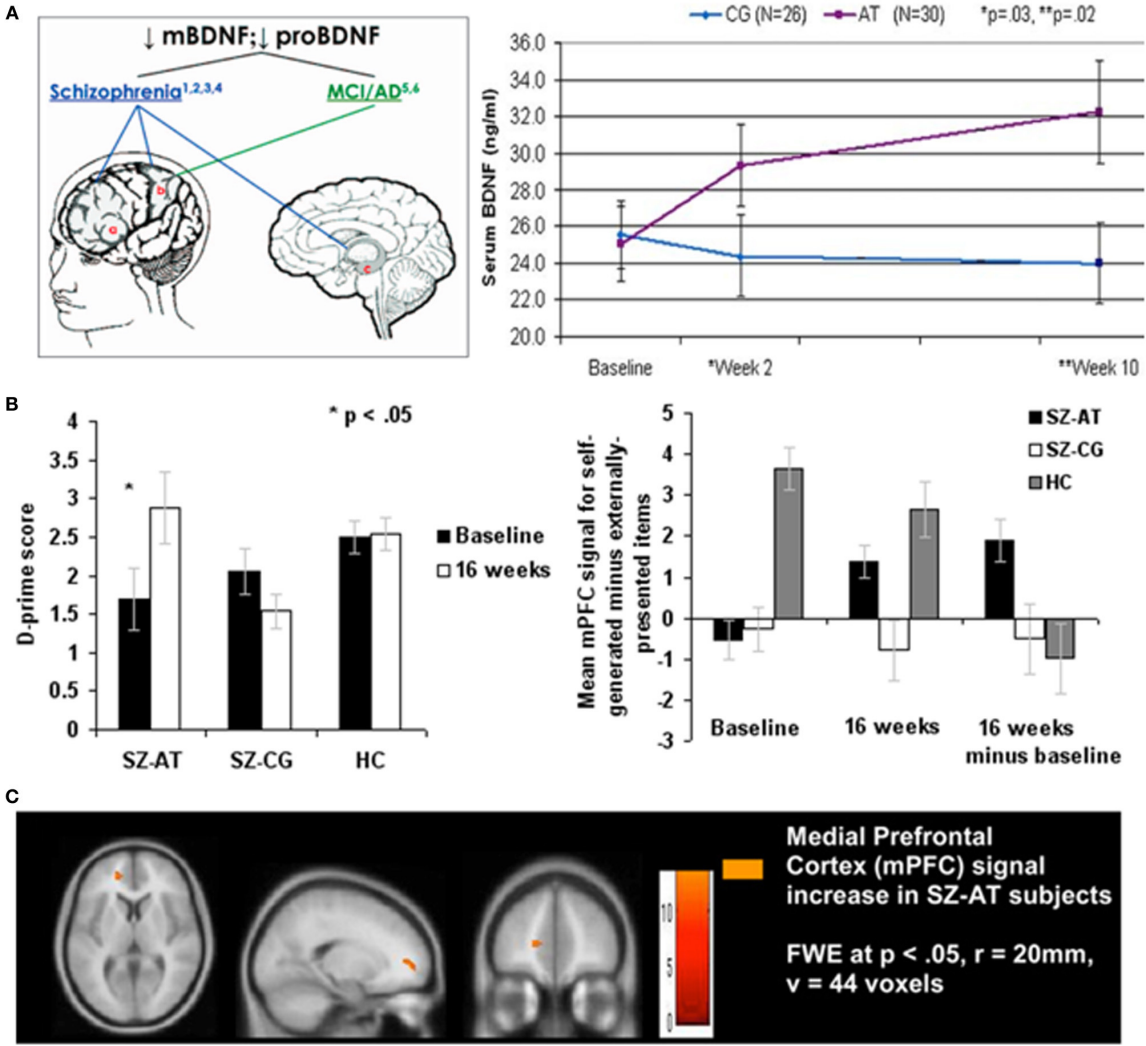
**Illustrating positive far-transfer chemical, behavioral, and brain response changes attributable to intensive brain training in patients with chronic schizophrenia (SZ)**. Tasks targeted the auditory-aural language/perceptual-cognitive system. Training was via computers, over a 40–50 hour-long training period. **(A)** Brain-Derived Neurotrophin Factor (BDNF) in its “pro” and mature (“m”) forms is down-regulated in schizophrenia and in other chronic neurological and psychiatric illnesses (e.g., healthy aging). As a result of this brain plasticity-based training, serum levels of BDNF were elevated to normal levels; no changes were recorded in subjects who worked with equivalent intensity on progressive control video games. The up-regulated of BDNF to near-normal levels was sustained for more than a year following training program completion. Similar effects have been recorded in aging brains. Data are from Vinogradov et al. (2009). **(B)** Left: Re-normalization of abilities in a behavioral task in which subjects with SZ identify whether they or an outside agent was the source of an immediate-past action. Again, this is a transfer effect of training; no aspect of this task is represented in the completed training regime. Right: Strengthening of BOLD responses in a medial prefrontal cortical area hypothesized to be the primary cortical site for the assignment of agency in the brain. No measurable task-related activity was recorded in this area in subjects with SZ prior to training, or in computer brain-engaged SZ controls before or after training. From Subramaniam et al. (2012). **(C)** Brain images showing changes in responses recorded in this task. Abbreviations: CG, computer games control; AT, auditory training; SZ-AT, schizophrenia patients in the auditory training group; SZ-CG, schizophrenia patients in the computer games group; HC, healthy controls.

Through collaboration with university-based scientists, different combinations of these forms of brain plasticity-based training have been applied in a large population of chronic schizophrenia and first-episode patients (e.g., Adcock et al., 2009; Fisher et al., 2009a,b; Dale et al., 2010; Popov et al., 2011; Subramaniam et al., 2012; Keefe et al., 2012; Sacks et al., 2013; see reviews by Biagianti and Vinogradov, 2013 and Fisher et al., 2013). For example, following 50 h of plasticity-based auditory training, chronic schizophrenia patients made significant gains in global cognition, processing speed, verbal working memory, and learning and memory metrics (e.g., Fisher et al., 2009a,b). In parallel, brains of trained subjects compared with controls recovered more normal M100 responses to successive signals consistent with recovery of more normal perceptual abilities resulting from training (Adcock et al., 2009; Dale et al., 2010); recovered more strongly correlated (recovered) gamma frequency responses in the lower gamma frequency (Popov et al., 2012); recovered stronger responses to rapidly successive stimuli in the gamma high-frequency domain (Dale et al., under review); more strongly synchronized alpha frequency responses for target stimuli, and more strongly de-synchronized non-target domain alpha-band responses in an attention-controlled task (Popov et al., 2012; Dale et al., under review); recovered more normal sensory gating (Popov et al., 2011); recovered more normal dorsolateral frontal responses in a working memory task (Dale et al., under review); restored more normal patterns of response in an attribution-of-source task (Subramaniam et al., 2012; see Figure 1); recovered more normal amygdala and ventral-lateral-frontal cortical responses in an emotion recognition task (Hooker et al., 2012, 2013); recovered more normal BDNF expression (Vinogradov et al., 2009); among other physical and functional neurological measures of plastic training-driven recovery.

While these studies are still a work in progress, taken together, they indicate that this form of computerized, neuroplasticity-based training is effective for broadly driving behavioral and physiological changes in a normal-ward direction in the schizophrenic brain. On this basis, we are now conducting a multi-site FDA medical device trial to further document these medical outcomes on the path to establishing medical claims for program use. Our longer-term goal is to progressively improve our training strategies to drive stronger and more complete and reliable changes in all key domains of dysfunction and loss in patients with this very complexly neurologically distorting illness.

It might be noted that given its bi-directional nature, training-driven plasticity processes are not riskless. Because we have a relatively complete understanding of the principles governing plasticity in our own medial therapeutic applications, we have not recorded negative consequences resulting from the application of any of our training tools. At the same time, an FDA approval process is important for assuring the affirmed positive values of the medical delivery of this new approach.

### Hemispatial neglect syndrome

Approximately half to two thirds of patients with right hemisphere injury exhibit a complex, debilitating array of spatial and non-spatial (attention) neurological deficits (Mesulam, 1990; Heilman et al., 1993). Those deficits arise from damage or disconnection to interconnected inferior parietal or lateral frontal cortical areas, or from the subcortical basal ganglia or thalamus. Patients with neglect do not respond to stimuli on the contra-lesional side of visual space, often seemingly unaware that anything in that space exists. For example, they may lose or fail to see or find objects located in neglected space, commonly suffer from poor navigation, and disregard significant events arising in the neglected field.

In addition to this manifest visual-field-localized impairment, patients with neglect exhibit deficits in attention that are not lateralized (Husain and Rorden, 2003; Van Vleet and Degutis, 2014). Those more general deficits are general in this population, and on that basis have been argued to be fundamental to the disorder (Corbetta and Shulman, 2011). They include deficits in arousal (particularly strong in the acute phase of recovery); attention to transient events; working memory updating; spatial working memory span; and alertness and sustained attention. Importantly, these non-spatial deficits are stronger predictors of chronic spatial neglect in the post-acute recovery phase than are the visuo-spatial deficits themselves (Hjaltason et al., 1996; Husain et al., 1997; Robertson et al., 1997; Duncan et al., 1999; Peers et al., 2006). Several recent studies show that the poor regulation of intrinsic alertness is correlated with—and plausibly explains—the degree of spatial field loss (“spatial bias”) in these patients (Robertson et al., 1997).

From a brain plasticity perspective, this is a particularly clear instance in which neuro-modulatory dysregulation contributing to attention control has been argued to be a strong contributor to disability. A pharmacological approach to recovery in such a case would be the administration of a stimulant drug; such drugs have been applied in this population with limited success (Fleet et al., 1987; Geminiani et al., 1998; Barrett et al., 2012). A cognitive behavioral approach would be to engage patients by heavily stimulating them to do what they can't do: respond to stimuli presented in the contralesional visual field. Again, that therapeutic approach has been applied with significant but limited success (Weinberg et al., 1977). Our neuroplasticity-based approach has applied training specifically designed to up-regulate both phasic and chronic alertness, combined with training designed to assure the recovery of more normal spatial working memory and representational salience for visual inputs arising from the affected visual field area. That approach is, again, predicated on our understanding of the normal modulatory processes in play. In the neglect patient, deficits in both tonic and phasic alertness are recorded. Tonic alertness (the background state of arousal) is highly correlated with the background level of expression of NA arising primarily from the midbrain LC (Sturm et al., 1999; Sturm and Willmes, 2001; Thiel et al., 2004). Tonic alertness is supported by a right-lateralized supra-modal network that feeds back to the LC, including the right inferior frontal, right inferior parietal and anterior cingulate regions. In contrast to the slowly changing tonic alertness, phasic alertness is the rapid modulation in alertness arising in any briefly engaging event, vital for operations such as orienting, selective attention, and the enabling of plasticity that is dependent upon these processes. This neuromodulation is largely attributed to the forebrain expression of ACh originating from the dorsal nucleus of Meynert, as well as by the phasic release of NA, again from the LC; their activation is again influenced by recurrent projections from a complex forebrain network. Phasic alertness is stronger against a platform of higher tonic alertness; both can be substantially amplified in an enduring way by relatively simple forms of training (DeGutis and Van Vleet, 2010; Van Vleet and DeGutis, 2013).

The primary strategy that we deploy to “exercise” this impaired neuromodulatory machinery is a continuous performance task in which subjects maintain a specific visual or auditory target in working memory (setting up the conditions for “top-down” neuromodulatory engagement), with those targets presented as rare events within a series of novel stimuli that are known to strongly activate limbic system sources of ACh (Richardson and DeLong, 1990; Sarter et al., 2001, 2006) and NA (Bouret and Sara, 2005). In the training task, subjects demonstrate that they are continuously attending to novel background stimuli by responding to them one by one; they demonstrate that they are holding a target stimulus in working memory by withholding their responding when it occurs (DeGutis and Van Vleet, 2010; Van Vleet and DeGutis, 2013). It might be noted that stimuli are also presented in this training in time-jittered sequences (see Wodka et al., 2009; Ryan et al., 2010) and that patients are progressively time-challenged in their responding to novel non-target stimuli (they must withhold responses for target stimuli), again because neurological studies indicate that this will drive more rapid and more enduring neurological remodeling.

**Figure 2:**
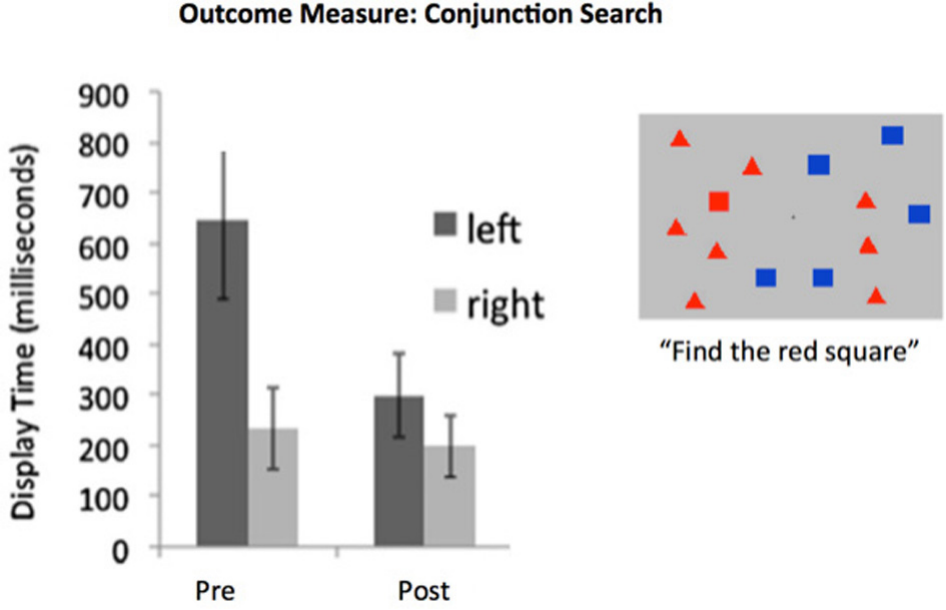
**Hemispatial neglect syndrome patients are slow to identify target stimuli arising in the neglected visual hemifield**. Training designed to recover the functionality of intrincsic alertness over a period of 6 h was adequate for achieving recovery when examined within 48 h post-training. Data from 20 neglect patients with a wide panoply of brain-injury etiologies. (e.g., tumor, stroke, TBI). Display time significantly shortened following training. Note that this is a “transfer” effect, as training involving the identification of objects, scenes, or tones was presented at central fixation (i.e., not in the neglected field). Redrawn from DeGutis and Van Vleet (2010) and Van Vleet and DeGutis (2013).

With the application of this neuro-modulatory- (attention-) targeted computerized training strategy alone, most neglect patients—including individuals with brain injury arising from almost any cause—rapidly recover their ability to “see” in the affected hemifield (Van Vleet and DeGutis, 2013; see Figure 2). Given this initial recovery, training is now more effectively extended to more completely recover representational fidelity and spatial working memory by directly re-refining the brain systems representing spatial and spatial sequencing and working memory deficits of neglect patients as well. A brain plasticity-based program based on these principles is now the subject of a large multi-site FDA medical device trial.

### Age-related impairment; resilience against neurodegenerative disease onset

By contrast to relatively sharply targeted training applied to address the problems that frustrate neglect syndrome patients, neurological and behavioral changes in aging, like those in schizophrenia, are almost brain wide. The documentation of age-related deficits—and the path of the progression to an ultimately catastrophic decline to senility—have been the subject of several hundred thousand scientific reports. The average aging brain expresses major progressively-growing behavioral deficits in all of its major processing systems in perception; speed of action and fluency; phasic, sustained and divided attention; different aspects of memory; social cognition; and executive, social and action control (see Salthouse, 2000, 2012; Reitz et al., 2011; Merzenich, 2013, for review). Losses translate to about an average of one third of a standard deviation per decade in ability after ability past the age of 30 for men, and beyond the age of about 45 in women. In neurological terms, substantial degradation is recorded in all great representational systems—again, on the average—in representational accuracy; processing speed; local response and system coordination; excitatory and especially inhibitory powers (and the complex machinery that support them); tracking of rapidly successive inputs; representation of temporal details of inputs(durations, intervals, rhythmic sequences, et al.); accurate representations of sequenced inputs, scenes, scenarios; the sustained responses supporting “working memory”/“selective attention”/“associative memory” and prediction; and the more complex “mental” neurological activities supporting executive, social and motor control, ideation, and thought (see Merzenich, 2013 and Nahum et al., 2013c, for review). All of these processes, and their degradation in aging, are again contributed to by parallel atrophy of the neuro-modulatory centers regulating the release of dopamine, norepinephrine, ACh, serotonin, et alia (Barili et al., 1998; Mufson et al., 2002; Backman et al., 2010)—which crucially support the plasticity processes that account for both functional maintenance and learning-based remodeling.

These neurological changes *are* “cognitive aging”: from a physical view, they are the basis of connectional dis-elaboration and disconnection, de-myelination, reduced blood flow, and neuropil and cortical shrinkage reduction attributable to dendrite, axonal arbor, glial process and synaptic simplification (e.g., see Mufson et al., 2002; Lo et al., 2011; Hahn et al., 2013; Jagust, 2013). Because of the broad picture of decline, both behaviorally and neurologically, there is a lot of re-engagement required to drive the brain broadly in a rejuvenating direction, conferring greater resilience re the onset of senile dimension and neurodegenerative diseases. At the same time, there is no simple pharmaceutical or cognitive behavioral strategy that could possibly drive the requisite, broad-scale corrections. The training program that we have designed to address these broad issues requires up to about 200 h to complete (dosing is dependent on the depth and breadth of neurological loss), with additional training on a lighter schedule required for many individuals to sustain a safe position over subsequent years. Again, all training is progressive and adaptive, and presented in game-like training formats on computers or other mobile devices. Our goal is to recover, insofar as possible, neurological representational accuracy, speed, coordination, sequencing, recording (remembering, selectively attending), noise control (distractor suppression) and executive processes in the brain. At the same time, exercises are designed to up-regulate, re-refine and re-invigorate modulatory control processes controlling learning, memory, attention states and mood.

As noted earlier, an important goal of this training is to increase resilience against the onset of neurodegenerative disease. Because we know the patterns of progression in pathology across a long epoch of time before frank “disease” onset, we increasingly understand how it relates to progressive changes in brain engagement. This understanding is also richly informed by the many factors that can accelerate the advance to Alzheimer's, Parkinson's and other neurodegenerative diseases (e.g., decreased noradrenergic activity; see Marien et al., 2004; Jardanhazi-Kurutz et al., 2010, 2011; Kong et al., 2010; McNamee et al., 2010; Koffie et al., 2011; Kalinin et al., 2012). We have extensively relied on this literature in the designs of programs to try to assure that training programs address the neurological, immunological, and vascular aspects of age-related impairment.

**Figure 3:**
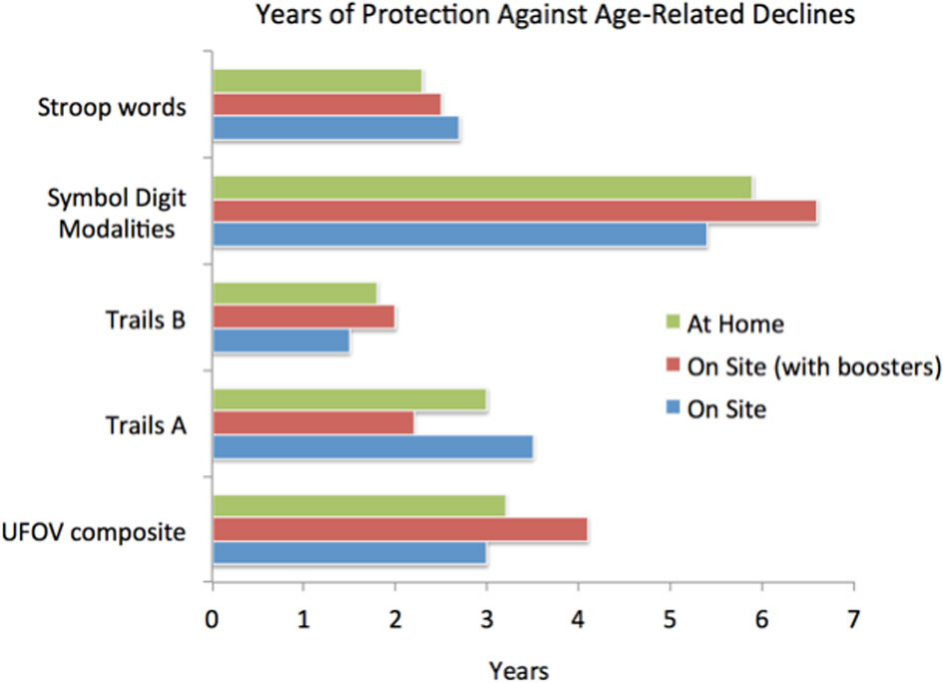
**Illustrating the magnitudes of gains for a limited computer delivered epoch of training (about 10 h) in a large (*n* = 670) cohort of healthy aged participants**. Training was conducted “at home” or in a clinical center at the University of Iowa. One population in the clinical center completed a 4-h “booster” training session 6 months after initial training program completion. All patients were behaviorally assessed before, immediately after, and 1 year after training program completion. Here, gains are expressed as an estimate of the number of years before assessment scores would be predicted to fall below pre-training scores; these highly significant gains had an average endurance of 3–4 years. Note that the “UFOV composite” reflects the approximately 1 *SD* gain in brain speed and visual control within an expanded visual field achieved directly from the training. All other measures represent near and far-transfer effects (i.e., benefits shown in untrained cognitive domains). Adapted from Wolinsky et al. (2013).

We have collaborated with university-based scientists who have conducted controlled studies in thousands of normally aging individuals to evaluate the effectiveness of this approach. Completed studies are still piecemeal, evaluating both the modality-specific and the general cognitive and neurological impacts of training in vision, hearing, executive control, attention, and related neuro-modulatory systems function. Studies of social cognition training are underway. All studies reveal a significant level of positive, enduring computerized training-driven improvements. To briefly summarize: (1) Training targeting the aural speech/language system have been shown to substantially improve measured listening, memory and related cognitive abilities, with significant far-transfer effects shown in quality of life/everyday life assessments (Mahncke et al., 2006; Smith et al., 2009; Zelinski et al., 2011). (Note that more than 250 additional studies demonstrating the behavioral and neurological values of this form of training have documented in studies in children and young adults. See Merzenich et al. (1998); and www.scientificlearning.com. In studies conducted in individuals of all ages, recoveries in perceptual abilities in listening have repeatedly documented rejuvenated speed of processing, accuracy, and attention control in processing abilities). (2) Training targeting visual perception and related cognition abilities resulted, in controlled trials, in significant improvements in visual processing (e.g., Ball et al., 2007; Berry et al., 2010; Wolinsky et al., 2013; see Figure 3). Improvements in speed and accuracy of processing and improvements in spatial vision (saccade sampling rates; multitasking; local and global reconstructions; scene reconstruction; useful field of view) were repeatedly recorded in these studies. These aural language and visual training studies also extensively documented improvements in attention, working memory, and immediate and delayed recall, and associative memory/syntactic abilities. (3) Studies document benefits of training for executive control and temporal and spatial navigation processes in training (e.g., see Ball et al., 2007; Smith et al., 2009; Merzenich, 2013). With working memory and with the highest levels of operation in social cognition, these explicit behaviors normally directly engage frontal, posterior parietal, anterior and posterior cingulate, medial ventral and hippocampal zones that undergo disconnection as a pre-amble to AD onset. (4) Broad far-transfer effects of training are recorded—e.g., to everyday quality of life (Ball and Edwards, 2007; Smith et al., 2009) to sustained confident independence (Edwards et al., 2009; Wolinsky et al., 2010a), to resilience impacts against the onset of depression (Wolinsky et al., 2009), to measures documenting improved brain health (Wolinsky et al., 2006, 2010b) and to sustained (Edwards et al., 2009) and safer automobile driving (Ball et al., 2010)—among other indices (Wolinsky et al., 2006, 2009, 2010a,b; Edwards et al., 2009; Ball et al., 2010). (5) Positive improvements have been shown to endure for many months to years following training completion (e.g., Wolinsky et al., 2006, 2009, 2013; Zelinski et al., 2011) (see Figure 3).

Does this form of training delay AD onset? Does it block, and can it reverse neuropathology progressions? Answering that question is the current goal of a large controlled internet-delivered trial currently underway. A growing body of evidence provides increasingly compelling evidence that this may, indeed, be the case. By training thousands of individuals at risk for AD onset, this question should be answerable, with finality, in the immediate future.

## Impediments for delivering these new treatment strategies to patients in need

The evolution of this new medical strategy for treating neurological and psychiatric illness is a “textbook example” of a disruptive technology. Its medicine is delivered via computers and other smart devices, at very low cost, without any requirement for the immediate presence of a medical professional. The scientific principles that support its use are poorly understood by most of the professionals who would normally prescribe and deliver this medicine. Most medical schools still focus on chemical and anatomical aspects of neuroscience on the path to creating pharmaceutically-focused medicine. Most graduate training in the psychology help professions still focus on “cognitive therapy” approaches to rehabilitation, with limited formal training in fundamental or integrative neuroscience on a level that informs this brain plasticity-based translational approach. Technological approaches are not the norm for a majority of practitioners in both of these large professional communities (see McMinn et al., 1999); even the minimum requirements in automated patient monitoring of compliance and progress potentially requiring professional participation and response delivered via the internet is beyond routine clinical practice for many specialists.

The delivery of these programs has also been confounded by a difficulty that the clinical community and public has in distinguishing between more classical cognitive therapeutic approaches delivered by computer from programs developed with application of a brain plasticity-based approach. Professional scientists have repeatedly described the limitations of the former—for example for achieving generalization beyond the directly trained tasks—describing their findings as demonstrating the limited values of any computer-delivered therapy. A result is great confusion for the professional community and public about the validity of all computer-delivered therapeutics in the brain health field.

Finally, the delivery of this form of medicine is impacted by the demands for compliance required of the patient. As we have noted earlier, driving the brain of an individual with schizophrenia or an individual at high risk for Alzheimer's Disease onset in a broadly re-strengthening direction can require many hours of intensive training potentially only achieved over a period of months. To substantially delay and to potentially achieve reliable prophylaxis against the onset of neurodegenerative disease, or to prevent schizophrenic onset, some almost-daily exercise may have to be undertaken for the rest of the patient's life. From a health perspective, the gains from this form of medicine often completely justify that effort; about 50–100 h to improve the level of cognitive ability for a patient with schizophrenia, for example (as was applied, for example, in Fisher et al., 2009a,b), is, after all, only about 1/100th the span of 1 year in their life. Clinical trials requiring this level of participation are now underway. Still, a public that is educated in ways that result in the broader patient acceptance of these forms of treatment as medicine is a key to their more successful, wider application.

Toward that end, the evaluation of program effectiveness through an FDA medical device process or its equivalent is a crucial part of our implementation strategy, and FDA-level trials are now underway for treatments designed to improve or recover the neurological status of patients with schizophrenia, traumatic brain injury, and stroke.

## An alternative vision of predominant future treatment modes in neurological and psychiatric medicine

As our understanding of our fundamental human neurology grows, the more we can expect it to be brought to bear as the basis of neurotherapeutic medicine. Up to this point, brain medicine has primarily followed a chemical therapeutics approach. When deficits are attributable to processes operating across complexly self-organizing brain systems whose functionality is impacted by several dozen major variables and is implemented through hundreds or thousands of gene-regulated chemicals, drug treatments necessarily have limited impacts that can rarely if ever be regarded as curative. Any “real cure” invariably requires complex brain rewiring that only the brain itself can achieve. This is the primary reason why there has been no major fundamentally new drug approved for use for more than 20 years. It is also the primary reason why addressing issues of aged infirmity that occur at an end stage of functional deterioration shall be a failure. In the end, only the brain, through its intrinsic plastic processes, can sustain or repair itself, at the level required to sustain high functionality. Those same good reasons explain that while pharmaceutical treatments can often rescue individuals with major neurologically and/or psychiatrically illness, the distortions manifested in their illnesses including the neurological alterations that are the basis of their disease routinely remain unaddressed (e.g., Opler et al., 2014).

In part because of the unsatisfactory clinical outcomes from chemical medicine, equally flourishing clinical practices try to address neurological impairment and psychiatric illness by documenting behavioral abnormalities as a premise for engaging the patient to directly address them. Again, the therapist commonly looks for a linchpin in behavior that can ameliorate the broader clinical symptoms. Because this strategy is usually removed from the underlying etiological causes of system distortions or failures in the brain, it again often fails to face up to the mechanistic realities that account for the clinical problems borne by the patient.

Brain plasticity-based therapeutics, still in its infancy, represents an attempt to address those real neurological distortions, on a level at which something closer to a fundamental neurological correction can potentially be achieved. As we understand how brain systems organize themselves, in detail, through their native plasticity processes, we understand with increasing clarity what plasticity itself has contributed to disease symptoms. Even more importantly, we understand how to harness these powerful intrinsic brain change processes to drive positive neurological corrections, on the path to something closer to a “cure.” Initial attempts to drive neurological correction on the requisite broad scale have usually generated still-incomplete brain remodeling; this translational science is still at a primitive stage, and very much a work in progress. At the same time, the brains of individuals with clinical conditions as complex as those that apply to the patient who is schizophrenic, is frail and struggling and at risk for collapse in aging, or has suffered from formerly-inexplicable visual field “blindness” following brain injury or stroke are clearly driven in a significantly improving—indeed, at least in most neurological respects re-normalizing—direction, by intensive computer-based training designed on these scientific bases. This form of medicine is inexpensive to deliver, as all it requires for operation is internet-connected tablet/computer/smart-phone (see Fernandez, 2011), and it is rapidly scalable for immediate application across the world.

We believe that this represents the advent of a new era in brain health medicine. In the future, we can expect to see a healthy re-integration of chemical, cognitive-behavioral, and brain plasticity-based therapeutic strategies. At the core, brain plasticity-based therapeutics can be expected to drive fundamental re-normalizing corrections for distorted brain systems. Those treatments shall be often supplemented in their actions by drugs or gene therapies that help patients overcome specific biological weaknesses assignable to genetic faults, or that augment the power of plasticity processes to accelerate positive neurological recovery. They shall also be supplemented by cognitive behavior approaches that more appropriately and more powerfully localize therapies to treat plasticity-induced distortions that spring from each patient's unique biographical history. With these novel therapeutics, treatment of the distorted chemical brain merges with treatment of the distorted behavior, on the grounds of brain plasticity based therapeutics.

### Conflict of interest statement

The authors all work for a for-profit company on the development of the therapeutic training programs. Those programs, and the science that supports their designs and uses, are described in this review.
